# Adverse events in patients with advanced urothelial carcinoma treated with erdafitinib: a retrospective pharmacovigilance study

**DOI:** 10.3389/fphar.2023.1266890

**Published:** 2023-11-21

**Authors:** Tengfei Yuan, Faping Li, Yuchuan Hou, Hui Guo

**Affiliations:** Department of Urology, The First Hospital of Jilin University, Changchun, China

**Keywords:** erdafitinib, FAERS, adverse event, data mining, pharmacovigilance

## Abstract

**Purpose:** On 12 April 2019, erdafitinib gained the first FDA approval as the second-line treatment for adult patients with locally advanced or metastatic urothelial cancer following progression during or after at least one previous line of platinum-based chemotherapy. However, the long-term safety profile of erdafitinib in a large patient population remains unexplored. The current study aimed to assess the adverse events (AEs) associated with erdafitinib through data mining of the US Food and Drug Administration Adverse Event Reporting System (FAERS).

**Method:** The reporting odds ratio (ROR), the proportional reporting ratio (PRR), the Bayesian confidence propagation neural network (BCPNN), and the multi-item gamma Poisson shrinker (MGPS) algorithms based on disproportionality were employed to quantify the signals of erdafitinib-associated AEs.

**Results:** A total of 6,322,279 reports of AEs were retrieved from the FAERS database spanning 2019 to 2022, out of which, 700 reports of erdafitinib as the “primary suspected” were identified. These erdafitinib-induced AEs were observed across 24 targeted system organ classes (SOCs). After conforming to the four algorithms at the same time, a total of 441 signals of erdafitinib-induced AEs were detected across 23 SOCs. Notably, signals associated with metabolism and nutrition disorders, eye disorders, and skin and subcutaneous tissue disorders were among the most prevalent. The median onset time for AEs was found to be 54 days [interquartile range (IQR) 17–112 days], with a majority of AEs occurring within the initial 6 months after initiating erdafitinib (37.23% within the first month, 15.53% within the second month, and 16.79% within the third month).

**Conclusion:** The findings of this study align with existing clinical observations, offering a comprehensive long-term post-marketing safety evaluation of erdafitinib. The results provide valuable evidence to enhance the understanding of erdafitinib’s safety profile, aiding further research and guiding clinical practice.

## Introduction

In 2023, bladder cancer (BC) stands as the seventh most prevalent malignant neoplasm in the United States, projecting an estimated 82,290 new cases and 16,710 fatalities (Siegel et al., 2023). Among the histological types, urothelial carcinoma (UC) represents the prevailing subtype, accounting for approximately 90% or more of BC cases (Bin Riaz et al., 2021). Unfortunately, about 30% of diagnoses manifest as muscle-invasive BC at the initial stage, predominantly presenting as locally advanced or metastatic disease, entailing a relative 5-year overall survival rate of merely 15% (Nadal and Bellmunt, 2019; Morales-Barrera et al., 2020).

Historically, cisplatin-based regimens have served as the first-line chemotherapy option for metastatic UC (mUC), revealing an overall response rate (ORR) of 50% and a median progression-free survival of 7 months (Roberts et al., 2006). However, almost half of the patients cannot undergo cisplatin chemotherapy due to factors such as renal impairment, inadequate treatment response, etc (Marandino et al., 2019). In recent years, the emergence of immune checkpoint inhibitors (ICIs) and fibroblast growth factor receptor inhibitors (FGFRs) has introduced novel therapeutic avenues for advanced UC management (Patel et al., 2020). FDA approvals have positioned ICIs as second-line systemic treatment for mUC. Although there is a durable response in some immunotherapy, many people will not benefit from immunotherapy (McConkey et al., 2016). Notably, gene expression profiling has facilitated the identification of specific UC subtypes (Robertson et al., 2017). For instance, the luminal I subtype exhibits reduced PD-L1 expression, rendering it less responsive to ICIs, whereas it manifests a higher FGFR3 mutation rate, including FGFR3 mutations and FGFR2/3 fusions (McConkey et al., 2015). Approximately 20% of patients with advanced UC exhibit alterations in FGFR, with FGFR2/3 gene fusion and mutation being the most prevalent, particularly in luminal I type, thus prompting downstream signaling pathway changes and instigating carcinogenesis (Haugsten et al., 2010; Knowles and Hurst, 2015). Consequently, FGFRs offer a promising therapeutic target for advanced UC treatment.

Erdafitinib, a potent and orally available FGFR one to four tyrosine kinase inhibitor (Garje et al., 2020), obtained its inaugural global approval by the FDA on 12 April 2019. It was indicated for treating adult patients with locally advanced or mUC harboring FGFR3 or FGFR2 genetic alterations, which progressed during or after at least one prior platinum-based chemotherapy, including cases within 12 months post-neoadjuvant or platinum-based adjuvant chemotherapy (Markham, 2019). The efficacy and safety of erdafitinib in this patient cohort were assessed in a multicenter, open-label, phase 2 study (BLC2001-NCT02365597) (Loriot et al., 2019). While generally well-tolerated as with other biological agents, erdafitinib exhibited some adverse reactions associated with its mechanism of action, dosage, or other factors. The most common AEs observed in the BLC2001 study included hyperphosphatemia (78%), stomatitis (35%), diarrhea (54%), nausea (22%), dry mouth (46%), dysgeusia (41%), fatigue (33%), nail changes (19%), elevated alanine aminotransferase (19%), and anemia (22%) (Peng et al., 2022).

It is essential to consider that prior investigations on erdafitinib were primarily based on clinical trials conducted under different specific conditions, and the AEs observed in such trials may not fully reflect all the AEs encountered in clinical practice (Fang et al., 2023). In addition, the limited sample size and follow-up time of these clinical trials may not adequately capture the full spectrum of observable AEs. More importantly, the onset time of adverse drug reactions (ADRs) associated with erdafitinib remains unknown. Therefore, exploring potential ADR signals linked to erdafitinib through data mining algorithms in large post-marketing samples is of paramount importance. The Federal Adverse Event Reporting System (FAERS) is a publicly accessible repository for spontaneous reports of post-marketing AEs submitted to the US FDA (Grace et al., 2020). As the largest pharmacovigilance database globally, FAERS serves as a highly effective tool for monitoring drug-related AEs. Therefore, the purpose of this study is to evaluate the AEs associated with erdafitinib via FAERS data mining, thereby providing valuable insights for future clinical safety monitoring and risk assessment endeavors.

## Materials and methods

### Study design and data source

**FIGURE 1:**
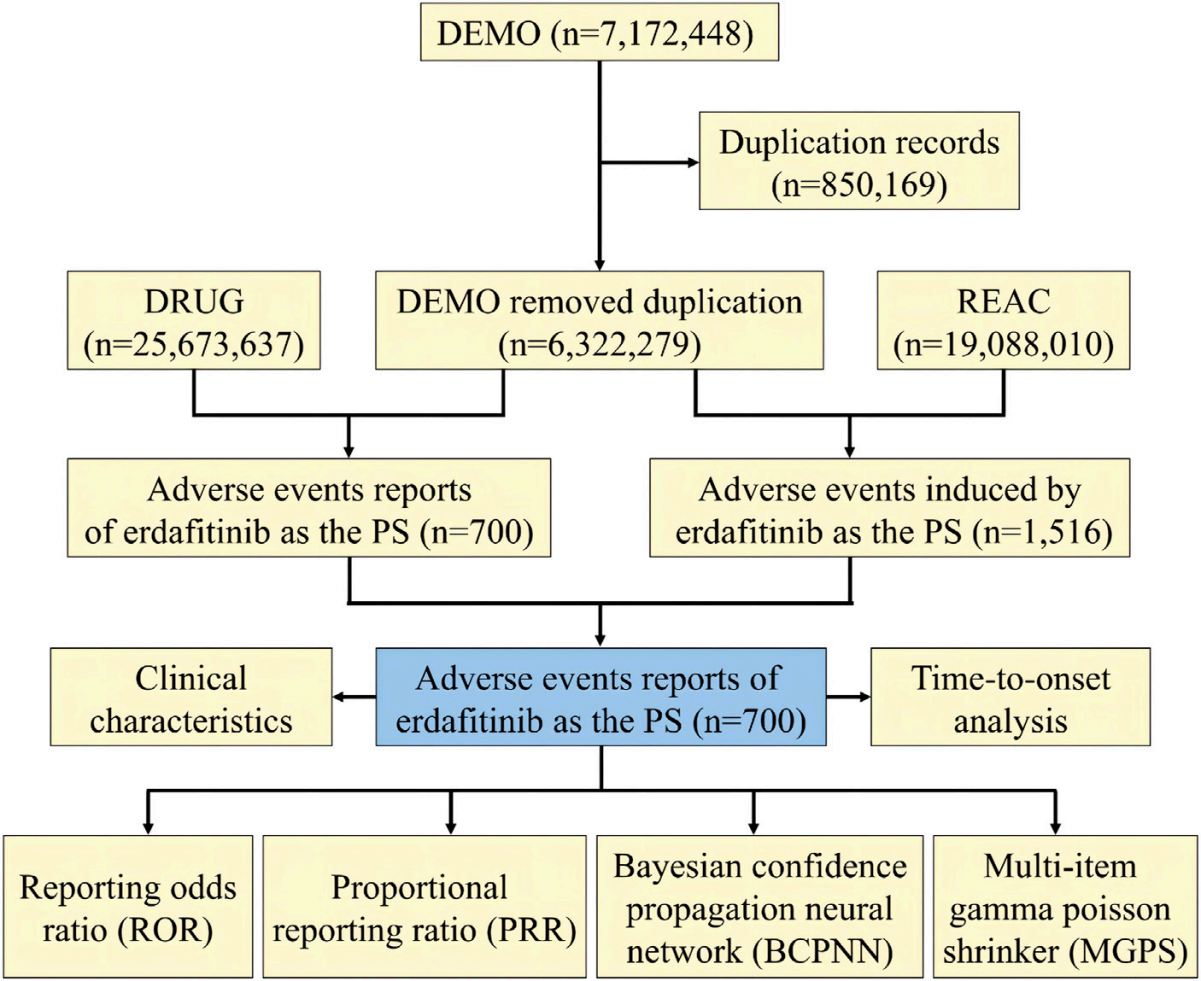
The process selecting associated-erdafitinib adverse events from FAERS.

We conducted a retrospective pharmacovigilance study based on the FAERS database, with data covering the period from 2019 to 2022. The FAERS data files contain seven types of datasets: patient demographic and administrative information (DEMO), drug information (DRUG), coded for the adverse events (REAC), patient outcomes (OUTC), report sources (RPSR), therapy start dates and end dates for reported drugs (THER), and indications for drug administration (INDI), and deleted cases (Yang et al., 2022). To facilitate statistical analysis, all data from the FDA were imported into MySQL software (v8.0; Oracle, Sweden), followed by a deduplication process (Shu et al., 2022b). Given that the FDA data receives submissions from various sources, potential duplicate reports needed reprocessing. In compliance with FDA recommendations, we selected the most recent FDA_DT when the CASEID was the same and opted for the higher PRIMARYID. By adhering to FDA guidelines and identifying instances where FDA_DT and CASEID were identical, we successfully eliminated duplicate reports originating from different individuals and institutions (Shu et al., 2022a). Consequently, the number of reports was streamlined, resulting in a total of 6,322,279 unique reports. A comprehensive flowchart outlining the meticulous processes of data extraction, processing, and analysis has been meticulously depicted in Figure 1.

### Adverse events and drug identification

The AEs reports available in the FAERS database have been meticulously coded according to the Medical Dictionary for Regulatory Activities (MedDRA) classification system. MedDRA’s structural hierarchy is organized into five hierarchical structures, namely, system organ class (SOC), high-level group term (HLGT), high-level term (HLT), preferred term (PT), and lowest-level term (LLT) (Mascolo et al., 2021). In the context of the current study focusing on associated-erdafitinib AEs retrieved from the FAERS database, both SOC and PT were standardized and classified to serve as the primary subjects for data analysis and research exploration. We identified cases in the DRUG files using generic names (erdafitinib in the drug name) and trade names (Balversa in the drug list) and selected role_cod as the PS to improve accuracy.

### Data mining

In the pursuit of pharmacovigilance studies, disproportionality analysis has gained wide acceptance. This approach compares the proportion of specific ADRs related to single or multiple drugs with the proportion of ADRs attributed to the same drug reported in the entire database (Hu et al., 2020). In our study, we used a disproportionality analysis to identify potential signals between erdafitinib and all AEs. The reporting odds ratio (ROR), the proportional reporting ratio (PRR), the Bayesian confidence propagation neural network (BCPNN), and the muti-item gamma Poisson shrinker (MGPS) algorithms are four major specific indices that were used to evaluate the signals of erdafitinib-related AEs (Guo et al., 2022). Among them, the ROR was utilized as one of the crucial indicators to evaluate the correlation between erdafitinib and AEs. ROR is a statistical measure utilized in pharmacovigilance to evaluate the association between a specific adverse event and a particular drug, in comparison to all other drugs in a database. In statistics, a higher ROR typically indicates a stronger association between the drug and the AE. The detailed computational equations and criteria for these four algorithms are shown in Table 1. Generally speaking, when the incidence of a specific AE related to erdafitinib significantly exceeds the background frequency within the database and reaches a certain threshold or standard, the higher the value of these four parameters, the stronger the signal (Yang et al., 2022).

**TABLE 1:** Four main algorithms are used to evaluate the association between erdafitinib and AEs.

Algorithms	Equation	Criteria
ROR	ROR = ad/b/c	lower limit of 95% CI > 1, N ≥ 3
	95%CI = e^ln(ROR)±1.96(1/a+1/b+1/c+1/d)^0.5^	
PRR	PRR = a(c + d)/c/(a+b)	PRR≥2, χ^2^ ≥ 4, N ≥ 3
	χ^2^ = [(ad-bc)^2](a+b + c + d)/[(a+b)(c + d)(a+c)(b + d)]	
BCPNN	IC = log_2_a(a+b + c + d)(a+c)(a+b)	IC025 > 0
	95%CI = E(IC) ± 2V(IC)^0.5	
MGPS	EBGM = a(a+b + c + d)/(a+c)/(a+b)	EBGM05 > 2
	95%CI = e^ln(EBGM)±1.96(1/a+1/b+1/c+1/d)^0.5^	

Equation: a, number of reports containing both the target drug and target adverse drug reaction; b, number of reports containing other adverse drug reaction of the target drug; c, number of reports containing the target adverse drug reaction of other drugs; d, number of reports containing other drugs and other adverse drug reactions. Note: 95%CI, 95% confidence interval; *N*, the number of reports; χ^2^, chi-squared; IC, information component; IC025, the lower limit of 95% CI, of the IC; E(IC), the IC, expectations; V(IC), the variance of IC; EBGM, empirical Bayesian geometric mean; EBGM05, the lower limit of 95% CI, of EBGM.

Furthermore, the onset time of erdafitinib-related AEs was calculated. Time to onset was defined as the interval between START_DATE (the start of erdafitinib use) and EVENT_DATE (the appearance of AEs). Measures were taken to exclude false and inaccurate AE occurrence information. MYSQL 8.0, Navicat Premium 15, Microsoft EXCEL 2019, and GraphPad Prism 8 were used for statistical processing and data analysis.

## Results

### Descriptive analysis

During the period spanning from 2019 to 2022, this study garnered a total of 6,322,279 reports from the FAERS database, diligently excluding duplicate data and procuring 700 AEs reports for erdafitinib as the PS (Figure 1). The clinical characteristics of events associated with erdafitinib were described in Table 2. Among these AEs, the proportion of males (51.57%) marginally surpassed that of females (33.71%). From the age distribution, the majority of patients experiencing AEs were aged over 65. A total of 232 (33.14%) death cases and 125 (17.86%) hospitalization cases were reported. Other serious consequences included life-threatening and disability. These AEs were primarily reported from five countries, with the United States (78.71%) accounting for the majority, followed by France (6.43%), Israel (2.29%), Spain (1.71%), and Brazil (1.57%). In addition, consumers (45.86%) and physicians (33.71%) were the main contributors in terms of reported occupations. In terms of reporting year, the highest number of AEs surfaced in 2020 (33.43%), followed by 2022 (31.43%), 2021 (26.43), and 2019 (8.71%).

**TABLE 2:** Basic characteristics of patients with erdafitinib-associated adverse events from the FAERS database.

Characteristics	Case number (n)	Case proportion (%)
Overall	700	
Gender		
Male	361	51.57
Female	236	33.71
Unknown	103	14.72
Age (years)		
<18	5	0.71
18–65	83	11.86
>65	184	26.29
Unknown	428	61.14
Outcome		
Death	232	33.14
Disability	8	1.14
Hospitalization	125	17.86
Life-threatening	9	1.29
Others	326	46.57
Reported person		
Health profession	92	13.14
Physician	236	33.71
Pharmacist	40	5.71
Consumer	321	45.86
Other health-professional	7	1.00
Unknown	4	0.57
Reported countries (top 5)		
United States	551	78.71
France	45	6.43
Israel	16	2.29
Spain	12	1.71
Brazil	11	1.57
Reporting year		
2019	61	8.71
2020	234	33.43
2021	185	26.43
2022	220	31.43

Disproportionality analysis signal strengths of AEs related-erdafitinib at the SOC level were described in Table 3. Statistically, AEs associated with erdafitinib were mainly distributed in 24 distinct system organs. The significant SOCs that satisfied at least one of the four calculation criteria encompassed Skin and subcutaneous tissue disorders, Eye disorders, Metabolism and infestations, General disorders and administration site condition, Gastrointestinal disorders, Surgical and medical procedures, and Hepatobiliary disorders.

**TABLE 3:** Signal strength of AEs of erdafitinib at the system organ class (SOC) level in FAERS database.

SOC	Cases	ROR (95% two-sided CI)	PRR (χ^2^)	IC (IC025)	EBGM (EBGM05)
General disorders and administration site conditions	380	1.57 (1.40–1.76)	1.43 (58.80)	0.51 (−1.15)	1.43 (1.27)
Gastrointestinal disorders	204	1.79 (1.55–2.08)	1.69 (62.14)	0.76 (−0.91)	1.69 (1.46)
Skin and subcutaneous tissue disorders	203	2.44 (2.10–2.83)	2.25 (149.53)	1.17 (−0.50)	2.25 (1.94)
Eye disorders	123	4.60 (3.82–5.53)	4.31 (318.30)	2.11 (0.44)	4.31 (3.58)
Injury, poisoning and procedural complications	105	0.57 (0.46–0.69)	0.60 (32.46)	−0.75 (−2.41)	0.60 (0.49)
Investigations	73	0.79 (0.63–1.00)	0.80 (3.75)	−0.32 (−1.98)	0.80 (0.63)
Nervous system disorders	67	0.58 (0.45–0.74)	0.60 (19.75)	−0.75 (−2.41)	0.60 (0.47)
Infections and infestations	62	0.73 (0.56–0.94)	0.74 (6.15)	−0.44 (−2.11)	0.74 (0.57)
Metabolism and nutrition disorders	59	2.04 (1.58–2.65)	2.00 (30.26)	1.00 (−0.66)	2.00 (1.54)
Respiratory, thoracic and mediastinal disorders	45	0.63 (0.47–0.85)	0.64 (9.35)	−0.64 (−2.30)	0.64 (0.48)
Musculoskeletal and connective tissue disorders	42	0.52 (0.38–0.71)	0.53 (18.23)	−0.91 (−2.58)	0.53 (0.39)
Neoplasms benign, malignant and unspecified (incl cysts and polyps)	39	0.59 (0.43–0.81)	0.60 (11.04)	−0.74 (−2.41)	0.60 (0.43)
Surgical and medical procedures	34	1.66 (1.18–2.33)	1.65 (8.75)	0.72 (−0.95)	1.65 (1.17)
Renal and urinary disorders	25	0.74 (0.50–1.10)	0.74 (2.28)	−0.43 (−2.10)	0.74 (0.50)
Vascular disorders	19	0.65 (0.42–1.03)	0.66 (3.47)	−0.61 (−2.27)	0.66 (0.42)
Hepatobiliary disorders	18	1.46 (0.92–2.33)	1.46 (2.61)	0.54 (−1.12)	1.46 (0.92)
Psychiatric disorders	8	0.09 (0.05–0.18)	0.10 (71.89)	−3.38 (−5.05)	0.10 (0.05)
Cardiac disorders	6	0.19 (0.09–0.43)	0.20 (19.94)	−2.34 (−4.01)	0.20 (0.09)
Blood and lymphatic system disorders	6	0.24 (0.11–0.52)	0.24 (14.86)	−2.07 (−3.74)	0.24 (0.11)
Ear and labyrinth disorders	5	0.80 (0.33–1.92)	0.80 (0.25)	−0.32 (−1.99)	0.80 (0.33)
Product issues	3	0.11 (0.04–0.35)	0.11 (21.15)	−3.14 (−4.81)	0.11 (0.04)
Immune system disorders	2	0.11 (0.03–0.42)	0.11 (15.22)	−3.23 (−4.90)	0.11 (0.03)
Reproductive system and breast disorders	1	0.10 (0.01–0.70)	0.10 (8.27)	−3.34 (−5.01)	0.10 (0.01)
Endocrine disorders	1	0.25 (0.04–1.78)	0.25 (2.24)	−1.99 (−3.66)	0.25 (0.04)

Upon simultaneous application of the four algorithms, a total of 441 signals corresponding to erdafitinib-induced AEs were detected, spanning 23 SOCs (Supplementary Table S1). The number of reporting PTs exceeding 10 was described in Table 4. Our study observed PTs encompassing hyperphosphatemia, stomatitis, diarrhea, nausea, dry mouth, fatigue, nail changes (disorder or discoloration), dry eye, onycholysis, decreased appetite, and taste disorder, which aligned with the documented AEs listed in the erdafitinib label. It is worth noting that since FAERS includes PTs that are related to medical and health, we also collected some signals that were not related to drugs, which mainly included injury, poisoning and procedural complications, and surgical and medical procedures.

**TABLE 4:** Signal strength of reports of erdafitinib at the preferred term (PT) level in the FAERS database. The number of reporting PTs >10 were showed.

SOC	PTs	Cases	ROR (95% two-sided CI)	PRR (χ^2^)	IC (IC025)	EBGM (EBGM05)
Eye disorders	Dry eye	21	18.48 (12.01–28.44)	18.24 (341.99)	4.19 (2.52)	18.22 (11.84)
	Eye disorder	14	17.63 (10.41–29.86)	17.48 (217.34)	4.13 (2.46)	17.46 (10.31)
	Visual impairment	11	3.48 (1.93–6.30)	3.47 (19.33)	1.79 (0.13)	3.47 (1.91)
Gastrointestinal disorders	Diarrhoea	41	2.62 (1.92–3.57)	2.57 (39.89)	1.36 (−0.30)	2.57 (1.89)
	Stomatitis	39	25.86 (18.81–35.55)	25.23 (906.57)	4.65 (2.99)	25.18 (18.32)
	Dry mouth	25	15.67 (10.55–23.28)	15.43 (337.41)	3.95 (2.28)	15.42 (10.38)
	Nausea	11	0.63 (0.35–1.14)	0.63 (2.37)	−0.66 (−2.33)	0.63 (0.35)
General disorders and administration site conditions	Death	205	11.36 (9.81–13.16)	9.97 (1675.99)	3.32 (1.65)	9.96 (8.60)
	Disease progression	22	8.32 (5.46–12.68)	8.22 (139.63)	3.04 (1.37)	8.21 (5.39)
	Adverse drug reaction	22	9.33 (6.12–14.22)	9.21 (161.18)	3.20 (1.53)	9.21 (6.04)
	Fatigue	19	0.97 (0.62–1.53)	0.97 (0.01)	−0.04 (−1.71)	0.97 (0.62)
	Mucosal inflammation	17	28.73 (17.80–46.36)	28.42 (448.90)	4.83 (3.16)	28.36 (17.57)
	Drug ineffective	16	0.44 (0.27–0.72)	0.45 (11.16)	−1.16 (−2.83)	0.45 (0.27)
	Adverse event	13	6.96 (4.03–12.01)	6.91 (65.71)	2.79 (1.12)	6.90 (4.00)
Injury, poisoning and procedural complications	Off label use	40	1.64 (1.20–2.24)	1.62 (9.63)	0.70 (−0.97)	1.62 (1.18)
	Product use in unapproved indication	18	1.99 (1.25–3.17)	1.98 (8.81)	0.99 (−0.68)	1.98 (1.25)
Investigations	Blood phosphorus increased	18	313.39 (195.80–501.62)	309.72 (5405.04)	8.24 (6.57)	302.24 (188.83)
	Weight decreased	14	2.03 (1.20–3.43)	2.02 (7.21)	1.01 (−0.66)	2.02 (1.19)
Metabolism and nutrition disorders	Hyperphosphataemia	18	458.09 (285.46–735.13)	452.72 (7829.02)	8.77 (7.10)	436.90 (272.25)
	Decreased appetite	13	2.34 (1.36–4.05)	2.33 (9.94)	1.22 (−0.45)	2.33 (1.35)
Musculoskeletal and connective tissue disorders	Pain in extremity	13	1.94 (1.12–3.34)	1.93 (5.85)	0.95 (−0.72)	1.93 (1.12)
Nervous system disorders	Taste disorder	12	14.26 (8.08–25.18)	14.16 (146.66)	3.82 (2.15)	14.14 (8.01)
Skin and sub-cutaneous tissue disorders	Onycholysis	25	684.76 (456.44–1027.28)	673.59 (15929.98)	9.32 (7.65)	639.13 (426.03)
	Nail disorder	23	124.90 (82.58–188.91)	123.04 (2757.17)	6.93 (5.26)	121.84 (80.56)
	Alopecia	17	3.02 (1.87–4.86)	2.99 (22.64)	1.58 (−0.09)	2.99 (1.86)
	Onychomadesis	17	229.60 (141.74–371.91)	227.06 (3757.85)	7.80 (6.13)	223.02 (137.68)
	Nail discoloration	16	175.69 (106.99–288.50)	173.87 (2712.30)	7.42 (5.75)	171.49 (104.43)
	Dry skin	15	3.26 (1.96–5.41)	3.23 (23.21)	1.69 (0.03)	3.23 (1.94)
Surgical and medical procedures	Hospitalisation	13	3.18 (1.84–5.50)	3.17 (19.31)	1.66 (−0.01)	3.17 (1.83)

### Onset time of events

**FIGURE 2:**
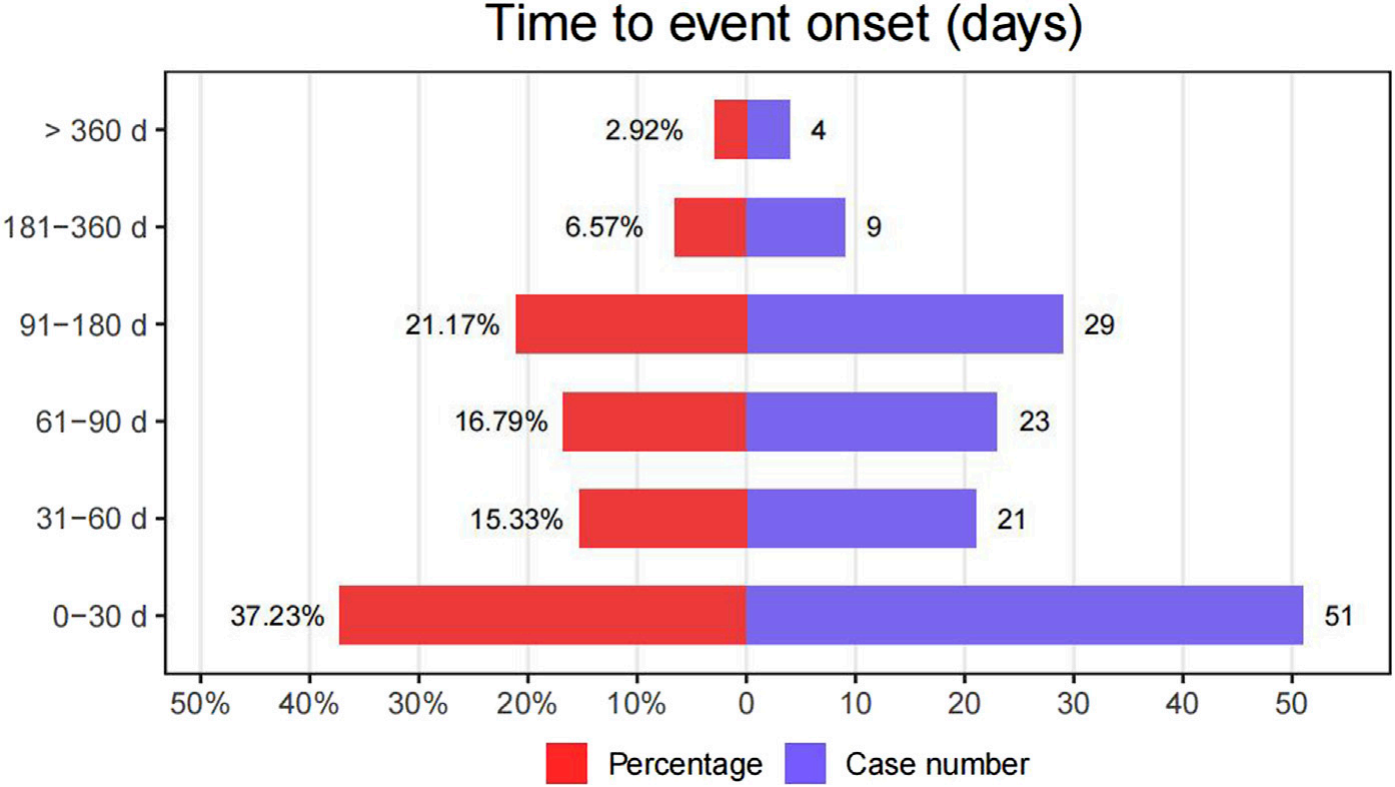
The onset time of AEs related to erdafitinib.

To discern the onset time of erdafitinib-associated AEs, data were meticulously collected from the database. Subsequent to the removal of duplicate and erroneous reports, a total of 137 AEs provided information on the onset time. The median onset time was calculated at 54 days [interquartile range (IQR) 17–112 days]. As illustrated in Figure 2, a substantial portion of the AEs manifested within the first (n = 51, 37.23%), second (n = 21, 15.53%), and third months (n = 23, 16.79%) after the initiation of erdafitinib treatment. It merits attention that after 1 year of erdafitinib treatment, 2.92% (n = 4) of AEs were still reported in our study.

## Discussion

Previous research on erdafitinib has primarily centered on clinical trials, mechanisms, and literature analysis, with limited emphasis on real-world investigations. To the best of our knowledge, this study is the first pharmacovigilance endeavor utilizing the FAERS database to explore potential associations between erdafitinib and AEs to evaluate the drug’s post-marketing safety. Our study results unveiled that, among the total reports examined, 361 instances (comprising 51.57% of the cohort) pertained to males, while 236 cases (constituting 33.71% of the cohort) pertained to females, both of whom exhibited erdafitinib-induced AEs. Notably, the occurrence of these AEs was more prevalent among males, potentially attributed to the elevated incidence of bladder cancer in the male demographic. This heightened prevalence led to a greater utilization of erdafitinib within the male population, consequently resulting in a larger male patient cohort within the purview of our investigation. Additionally, a higher proportion of AEs was observed among individuals aged over 65, warranting vigilant monitoring of AEs in elderly male patients. The early identification of AEs holds critical importance, as these events may pose life-threatening risks or contribute to disease progression.

According to disproportionation analysis, at the SOC level, the most common and meaningful signals were eye diseases, metabolic and nutritional diseases, and skin and subcutaneous tissue disorders. Hyperphosphatemia, skin and nail changes, and eye disorders were identified as typical ADRs of EFGR inhibitors. In phase I and phase II clinical trial, the above AEs were also confirmed to have occurred, which was consistent with our results (Marandino et al., 2019). Eye diseases were recurrently observed in the drug label of erdafitinib, with dry eye, eye diseases, and visual impairment comprising the most frequently reported AEs within the SOC of ocular diseases. In I and phase II studies, dry eye, and blurred vision were observed in almost more than half of the patients (Loriot et al., 2019). Our findings affirm the prevalence of dry eye as the most common ocular abnormality, and the signal intensities measured as ROR 18.48 (12.01–28.44), PRR 18.24 (341.99), IC 4.19 (2.52), and EBGM 18.22 (11.84), respectively. Besides, approximately 25% of patients exhibited central serous retinopathy or retinal pigment epithelium detachment (D'Angelo et al., 2020). Studies have found that ocular toxicity, including central serous retinopathy, is the class effect of known mitogen-activated protein kinase pathway inhibitors (Loriot et al., 2019), potentially related to erdafitinib’s inhibition of FGFR-related downstream pathways. Normally, central serous retinopathy is temporary, and visual impairment will improve gradually following appropriate dose reduction or discontinuation. Therefore, as a precautionary measure, baseline ophthalmic examination should be repeated monthly during the initial 4 months of erdafitinib treatment, followed by evaluations every 3 months (Sayegh et al., 2022).

In addition, metabolism and nutritional diseases, particularly hyperphosphatemia and decreased appetite, emerged as other important and non-negligible AEs signals related to erdafitinib. Hyperphosphatemia is a recognized type of toxicity attributed to FGFR inhibitors and has been reported in about 77% of patients (Montazeri and Bellmunt, 2020). In our study, despite the relatively few reports on hyperphosphatemia (18), it exhibited a significant signal intensity, with ROR 458.09 (285.46–735.13), PRR 452.72 (7829.02), IC 8.77 (7.10), and EBGM 436.90 (272.25), respectively. Studies have found that FGFR regulates the excretion of phosphate in serum in renal tubules, and inhibition of the FGFR pathway in the renal tubules can lead to hyperphosphatemia (D’Alessandro et al., 2015). Subsequently, a correlation study between erdafitinib plasma concentration and serum phosphate levels demonstrated a significant association (Bahleda et al., 2019). In a further phase I study (NCT01962532) involving Japanese patients with advanced or refractory solid tumors, a single oral dose of 2, 4 and 6 mg erdafitinib was associated with increased plasma phosphate concentration, but no clear dose-response relationship was observed (Markham, 2019). Thus, it is recommended that patients taking erdafitinib should adhere to a dietary phosphate intake of 600–800 mg per day, diligently monitor serum phosphate levels on time, and discontinue treatment if necessary.

Another noteworthy observation pertains to the long-term use of erdafitinib, which carries the risk of dermal AEs, with onycholysis, nail discoloration, alopecia, and dry skin being the most prevalent within the skin and subcutaneous tissue diseases. In a multicenter phase I trial of erdafitinib (JNJ-42756493), 43% of patients experienced skin changes, with dry skin being the most common (29%), and 33% of patients reported nail changes, with onycholysis (11%) and nail dystrophy (9%) being the most frequent occurrences (Bahleda et al., 2019). In this study, onycholysis exhibited the highest number of reported cases in the category of skin and subcutaneous tissue diseases and showed a strong correlation with signal intensity, indicated by ROR 684.76 (456.44–1027.28), PRR 673.59 (15929.98), IC 9.32 (7.65), EBGM 639.13 (426.03), respectively. These findings align with the outcomes reported in the aforementioned clinical trials. However, the underlying pathophysiological mechanisms responsible for these dermal-related AEs have yet to be conclusively determined (Lacouture et al., 2021). Several possible pathological mechanisms have been proposed, such as erdafitinib-induced inhibition of the FGFR pathway in keratinocytes, which may lead to disorders in hair follicle homeostasis and epidermal proliferation and/or differentiation, alongside downregulation of tight junction gene expression, as demonstrated in FGFR-deficient mice (Yang et al., 2010). Therefore, the occurrence of skin and subcutaneous tissue disorders constitutes a significant event necessitating due attention.

Moreover, gastrointestinal diseases also manifested common AEs on the label of erdafitinib, with dry mouth and stomatitis, respectively (Bahleda et al., 2019), among them, dry mouth, typically observed at grade 1 or 2, which was common in patients treated with FGFR inhibitors, occurred in approximately 31%–46% of patients with UC (Lacouture et al., 2021). As evidenced by our study, the dry mouth was detected as obvious signal strength being ROR 15.76 (10.55–23.28), PRR 15.43 (337.41), IC 3.95 (2.28), and EBGM 15.42 (10.38), respectively. Research has found that FGF and FGFRs play a central role in the morphogenesis of salivary gland branches where the destruction of these factors or their receptors probably has been shown to have an impact on salivary gland function, causing the performance of dry mouth (Prochazkova et al., 2018). In addition, in our study, death-related AEs showed obvious signals, which were rare in erdafitinib-related AEs and may be related to the patient’s disease progression rather than the drug itself.

Despite the existence of clinical trials and normative reports, patients receiving erdafitinib treatment also showed hematological abnormalities, especially decreased hemoglobin (35%), decreased platelet count (19%), decreased white blood cells (19%), and neutrophils (10%) (Roubal et al., 2020). However, these AEs did not appear as significant signals in our data analysis. Similarly, several common AEs listed on the drug label, such as hand and foot syndrome, abnormal liver function (including elevated ALT and AST), diarrhea, or constipation, were not detected as signals in our study. These phenomena can be explained by the fact that AEs of all drugs in the FAERS database are quite common. The signal scores can be attenuated due to a high volume of reports for AEs related to various drugs (Fang et al., 2023). Disproportionation analysis requires a higher (or lower) frequency of AE reporting for a particular drug. The absence of signals does not imply the absence of correlation AEs but rather indicates that these AEs do not exhibit a disproportionate relationship (Markham, 2019; Fang et al., 2023).

This research indicated that the median onset time of erdafitinib-induced AEs following initial treatment was 54 days [(IQR) 17–112 days], with a majority of AEs occurring within the first (n = 51, 37.23%), second (n = 21, 15.53%), third months (n = 23, 16.79%). These findings were consistent with the previous report in an experimental environment where 27% of patients developed symptoms of central serous retinopathy at 53 days (15% of patients taking 8 mg per day, 12% of patients taking 9 mg per day) (Peng et al., 2022). However, it should be noted that our study included only 137 AE reports with recorded onset times, potentially limiting the accurate reflection of actual onset times and warranting further verification. Therefore, it is essential for clinicians to be vigilant about the onset time, proactively identify and prevent AEs, and promptly implement effective measures.

At present, there is a scarcity of studies focusing on the safety of erdafitinib in real-world large samples. In this regard, our research is particularly noteworthy as it represents the first large-scale investigation into post-marketing AEs of erdafitinib based on the FAERS database. By conducting data mining on the FAERS database, our study systematically explored and analyzed the common signals of adverse reaction to erdafitinib, such as hyperphosphatemia, dry mouth, and eye diseases, as well as other meaningful AEs reports and their respective onset times. This research contributes valuable insights into the clinical safety profile of erdafitinib for future reference.

However, there are still some limitations in our research. First of all, due to the FAERS database relies on a spontaneous reporting system, gathering information from different countries and occupations, which may lead to issues such as underreporting, and incomplete or inaccurate reports, thus potentially affecting the robustness of our research findings. Therefore, some degree of deviation in the result was expected. Secondly, several unmeasured confounding factors, such as potential drug-drug interactions and patient comorbidities that could impact AEs, were not accounted for in our data analysis. Thirdly, it is crucial to emphasize that disproportionality analysis, although valuable in assessing signal intensity, does not provide quantitative risk or prove causal relationships between AEs and targeted drugs. Nevertheless, the FAERS database remains an important tool for post-marketing safety surveillance of drugs.

## Conclusion

This study utilized the comprehensive FAERS database, spanning from 2019 to 2022, to acquire a total of 6,322,279 reports after removing duplicates, and successfully identified 700 AE reports associated with erdafitinib as the PS. Through the application of disproportionality analysis, erdafitinib-related AE signals were rigorously explored and quantified, encompassing the onset time and safety signal spectrum of AEs. Notably, common AEs identified at the SOC level included hyperphosphatemia, eye diseases, and dry mouth. It is worth mentioning that the AEs reported in this study demonstrated a considerable concordance with the previously reported clinical trial outcomes and the erdafitinib label provided by the manufacturer. This extensive post-marketing safety surveillance significantly contributes to a more profound comprehension of erdafitinib’s safety profile, thus offering valuable evidence to inform future research and clinical practice in the field.

## Data Availability

The datasets presented in this study can be found in online repositories. The names of the repository/repositories and accession number(s) can be found in the article/Supplementary Material.
